# Examining the Moderating Role of Patient Enablement on the Relationship Between Health Anxiety and Psychosomatic Distress: A Cross-Sectional Study at a Traditional Chinese Medicine Outpatient Clinic in Hong Kong

**DOI:** 10.3389/fpsyg.2020.01081

**Published:** 2020-06-09

**Authors:** Celia H. Y. Chan, Bobo H. P. Lau, Timothy H. Y. Chan, H. T. Leung, Georgina Y. K. So, Cecilia L. W. Chan

**Affiliations:** ^1^Department of Social Work and Social Administration, The University of Hong Kong, Hong Kong, China; ^2^Department of Counselling and Psychology, Hong Kong Shue Yan University, North Point, Hong Kong

**Keywords:** health anxiety, patient enablement, psychosomatic distress, primary care, Chinese medicine

## Abstract

**Background:**

Little research effort has been devoted to examining the role of patient enablement in alleviating health anxiety in primary care. In this study, we examined the role of patient enablement as a moderator in the relationship between health anxiety, psychological distress, and treatment seeking in traditional Chinese medicine (TCM).

**Methods:**

The participants were 634 patients of a government-subsidized Chinese medicine outpatient clinic in Hong Kong. They were asked to complete a series of questionnaires on patient enablement, health anxiety, anxiety, depression, physical distress, annual clinic visits, and service satisfaction and provided various demographic details. Descriptive statistics, correlations, and general linear models were used to analyze the data.

**Results:**

We found that patient enablement correlated positively with service satisfaction. Patient enablement also interacted significantly with health anxiety in affecting indices of psychological distress (depression, anxiety) and treatment seeking (annual visits). Among highly enabled patients, the positive association between health anxiety and indices of psychological distress was weakened, and they also showed more health anxiety–driven treatment seeking as measured by annual clinic visits.

**Conclusion:**

These findings suggest a moderating mechanism by which patient enablement weakens the relationship between health anxiety on psychological well-being and increases treatment-seeking behavior in TCM. Practitioners are encouraged to provide sufficient information to patients to foster self-care and disease self-management using complementary and alternative medicine (CAM).

## Introduction

Health anxiety is defined as a range of worries and preoccupations regarding illness and pain, which respond readily to appropriate medical reassurance (Sirri and Fava, 2013; Fava et al., 2017). Prevalence rates of health anxiety were found to range from 2 to 13% in population studies (Gropalis et al., 2012; Weck et al., 2014; Scarella et al., 2019). The conceptualization of health anxiety has undergone substantial refinement over the past decades. Notably, the Diagnostic Criteria for Psychosomatic Research (DCPR) is the first diagnostic framework to explicitly characterize health anxiety as a type of “abnormal illness behavior,” which manifests as general preoccupation about health regardless of whether a health problem is actually present (Sirri and Fava, 2013). The construct of health anxiety as used in this study, thus, follows the conceptualization of Fava et al. (2017) as a type of illness behavior that is different from hypochondriasis and disease phobia by virtue of it being less specific and more responsive to medical reassurance than the latter categories.

Theoretically, health anxiety and somatic distress appear to be connected by maladaptive cognitive-psychological processes, namely the selective and focused attention to signs of bodily discomfort and interpretation of these symptoms as signs of physical ill health. According to this cognitive-psychological framework, the more convinced one is about developing an illness, the more motivated one would be to maintain attention to and exaggerate the perception of bodily symptoms, leading to further catastrophizing (Brown, 2004; Witthöft et al., 2016). This is supported by empirical evidence pointing to a link between health anxiety and somatic symptom distress among clinical (Fergus et al., 2018) and non-clinical populations (Lee et al., 2015). Similarly, the presence of psychological symptoms, such as depression and anxiety, may prompt selective attention to bodily sensations, which, in turn, may trigger suspicion of ill health (Sirri et al., 2015; Oliver et al., 2018). That health anxiety is uniquely associated with psychosomatic distress is corroborated by findings of an empirical relationship between health anxiety and rate of healthcare utilization (Weiss et al., 2017; Horenstein et al., 2019). Nevertheless, studies that expounded on the theoretical relationship between health anxiety and psychosomatic distress often adopt varied definitions of health anxiety, many of which share considerable overlap with similar yet conceptually distinct disorders, such as hypochondriasis and illness anxiety disorder. In face of this conceptual limitation, this theoretical relationship is yet to be further explored with a better defined construct of health anxiety.

An early study conducted by Noyes et al. (2003) found a weak-to-modest negative correlation (*r* = 0.2–0.3) between the quality of patient–doctor encounter and health anxiety, suggesting there is an interpersonal element to health-related anxiety. An interpersonal model of health anxiety, which views health anxiety as a maladaptive behavioral manifestation of attachment insecurity developed from one’s early years, has found increasing empirical support in recent years (MacSwain et al., 2009; Birnie et al., 2013; Sherry et al., 2014). Given that people with high health anxiety are more likely to seek help from medical professionals, many studies on health anxiety were conducted in primary care settings (Robbins and Kirmayer, 1996; Gureje et al., 1997; Escobar et al., 1998; Toft et al., 2005; Hanel et al., 2009). Existing studies focused primarily on the diagnosis, prevalence, trajectory, psychiatric comorbidity, and demographic correlates of health anxiety as well as the personality styles and resource utilization of individuals exhibiting health anxiety. Yet there is a lack of investigation on the interpersonal aspect of health anxiety in primary care settings. Previous studies have revealed individuals with high levels of health anxiety tend to have higher service usage (Lee et al., 2015), and those who have received more patient-centered care tend to use fewer healthcare services (Bertakis and Azari, 2011). Number of annual visits was, therefore, added alongside bodily distress as an outcome to reveal the effects of patient enablement and health anxiety on healthcare resource utilization.

The effectiveness of primary healthcare is often assessed in terms of how satisfied patients are with the clinical consultation conducted by the doctor. In continuity with the notion of patient-centered care, the concept of patient enablement is developed to capture more specifically the extent to which patients feel empowered to cope with, understand, and manage their condition after attending the consultation, one that is distinct from the concept of patient satisfaction, which focuses on the effectiveness of the clinical consultation itself (Howie et al., 1998; Hudon et al., 2010; Desborough et al., 2017; Barratt and Thomas, 2019). The concept of enablement is especially relevant in primary care settings, where people tend to seek general consultations as a result of problems that have a combination of physical, psychological, and social elements (Howie et al., 2004; Lam et al., 2010). In practice, patient enablement is viewed as the outcome of the clinician exhibiting patient-centered practices, empathic communication, and other interpersonal factors, such as the continuity of care (Frost et al., 2015). In a sample of adult patients with chronic musculoskeletal disorders recruited form primary care, Enthoven et al. (2019) found unique associations between patient enablement and physical (i.e., disability) and mental health outcomes (i.e., depression, anxiety). Similar results were noted in patients utilizing CAM, such as acupuncture, homeopathy, and sophrology (Gaitan-Sierra and Hyland, 2015). However, in studies exploring the relationship between patient-centered care and health outcomes, the concept of enablement is often not clearly differentiated from the concept of individual empowerment, which refers more generally to the process of exercising control and decision making over one’s health (Hudon et al., 2010). In this study, we look specifically at the role of enablement in the relationship between health anxiety and physical and psychological health outcomes.

The impact of patient enablement was studied under the setting of a TCM outpatient clinic in the current research. Compared to Western medicine, TCM relies on a different representation of health. In TCM, health is construed as a state of dynamic balance across various interrelated faculties of human existence, including the biological, psychological, and spiritual (Chan et al., 2002). To achieve this dynamic balance, people are advised to be authentic (*ziran*), follow through the natural course of action, and yield to the flow of immediate circumstances (*wuwei*). These states can be achieved through cultivating healthy habits, such as eating right, exercising right (e.g., practicing Qigong), taking rest at appropriate times, maintaining equanimity, avoiding afflictions, living a simple life, etc. These health-enhancing habits are central to the pursuit of health according to TCM but are mostly within the reach of patients and can be well blended into patients’ daily lives (Chung et al., 2014). In contrast, Western medicine, which does not necessarily embrace a holistic concept of care, tends to conceptualize ill health as being due to the invasion of external, foreign objects (e.g., virus, bacteria), which has to be exterminated through professionally dispensed drugs and procedures. The role of patients in traditional Western medicine without a holistic concept of health could be as a mere recipient of professional service rather than an active manager of one’s health. One of the objectives of patient enablement is to address this very issue.

To the best of our knowledge, no study has specifically looked at the relationships between patient enablement, health anxiety, and psychosomatic distress in primary care settings. Health anxiety has well-established links with physical and psychological well-being (Fergus et al., 2018; Oliver et al., 2018). As suggested by the interpersonal model of health anxiety, interpersonal factors, such as the clinician–patient encounter may be uniquely related to the overall well-being of patients, especially given that health-related reassurance seeking is a dominant behavior exhibited by health-anxious individuals (Birnie et al., 2013). Given the dearth of research in this area, the current study was set out to answer the following question: Does patient enablement moderate the effects of health anxiety on psychological and physical well-being in primary care patients? Specifically, we hypothesize that patient enablement moderates the relationship between health anxiety and physical and psychological distress experienced by primary care patients attending a TCM clinic.

## Materials and Methods

### Participants

Adults aged 18 or above were recruited at a government-subsidized TCM outpatient clinic under the public healthcare system in Hong Kong, whose population of 305,100 residents had a median age of 40 and median monthly household income of HKD 18,000 (USD 2,307). Since its return to Chinese sovereignty in 1997, the Hong Kong government has taken measures to recognize and institutionalize TCM, resulting in the introduction of a TCM regulatory body and the opening of government-subsidized TCM outpatient clinics as an integral arm of the primary care system. TCM consultations in Hong Kong were found to be as effective as general consultations in Western medicine in terms of its impact on quality-of-life outcomes (Wong et al., 2011). The clinic was open to all local residents.

This cross-sectional study was conducted between 2014 and 2015. A reception was set up daily inside the clinic’s waiting area during the study period, and all patients who met eligibility criteria were invited by volunteer healthcare workers at the clinic to participate in the study (i.e., consecutive sampling). Eligible participants were approached and received information concerning the purpose and procedure of the study. After giving written consent, participants completed measures of health anxiety, psychosomatic distress, and patient enablement in the clinic waiting area. After participants consulted with the TCM practitioner, they were asked to rate the level of satisfaction toward the consultation and indicate the number of visits they paid to the doctor in the past year. They received a patient booklet on mental well-being as a gift after the completion of the survey. This study was approved by the Institutional Review Board of The University of Hong Kong/Hospital Authority Hong Kong West Cluster (UW 13-088). All participants gave written consent prior to participating in the study.

### Measures

#### Health Anxiety

Health anxiety was measured by the Whiteley-7 Index (Fink et al., 1999), a list of seven binary-choice items that measure the extent of health anxiety, encompassing illness somatization and tendency for hypochondriasis, such that higher scores reflect greater health anxiety. For reference, items include “Do you worry a lot about your health?” and “Do you often worry about the possibility that you have a serious illness?” It has been shown to be an effective instrument to differentiate hypochondriacal and non-hypochondriacal patients (Speckens et al., 1996). The instrument was translated to traditional Chinese from the original English version and validated in the Hong Kong population by Lee et al. (2011), who, consistent with past literature (Fink et al., 1999; Conradt et al., 2006), found a one-factor structure of the measure in their sample. Kuder-Richardson 20 (KR20) for the Chinese scale was 0.73 (Lee et al., 2011).

#### Psychosomatic Distress

Psychosomatic distress was measured by three instruments: Physical distress was measured by the Body-Mind-Spirit Well-being Inventory (BMSWBI)–Physical Distress subscale, which has demonstrated satisfactory reliability with a Cronbach’s α of 0.87 in a Chinese community sample (Ng et al., 2005). The Physical Distress subscale measures the severity of 14 somatic symptoms (e.g., headache, insomnia, constipation, back pain, etc.) that are common among the Chinese population on a scale of 0 (no distress at all) to 10 (extreme distress). A higher summative score on the scale denotes greater physical distress. Psychological distress was measured by two instruments: Patient Health Questionnaire (PHQ-9) (Kroenke et al., 2001) and General Anxiety Disorder 7-item Scale (GAD-7) (Spitzer et al., 2006). Both are well validated and widely used brief instruments that measure the severity of depression and anxiety, respectively. The traditional Chinese versions used in this study demonstrated satisfactory reliability with a Cronbach’s α of 0.86 for PHQ-9 (Wang et al., 2014) and 0.90 for GAD-7 (He et al., 2010).

#### Patient Enablement

Patient enablement was measured by the 6-item Patient Enablement Instrument (PEI) developed by Howie et al. (1998), which asks respondents to report their ability to cope with life, ability to understand and cope with their own illness, ability to maintain good health, confidence in own health, and ability to help oneself after consulting with a clinician. Items were rated on a scale of 0 (the same or less) to 2 (greatly improved), such that higher scores correspond to greater enablement. The Chinese version used in this study had a reliability of 0.85 (Lam et al., 2010).

#### Service Usage and Satisfaction

To measure service satisfaction, a 6-point Likert item was adapted from Lee and associates (Lee et al., 2011): “Are you satisfied with the Chinese medicine practitioner at the clinic?” (0 = not satisfied at all; 5 = very satisfied). The participants were also asked to indicate the number of visits paid to the TCM clinic in the past year.

### Statistical Analysis

Data was summarized by descriptive statistics. Pearson correlation coefficients were calculated between each pair of variables of interest. The moderating role of patient enablement on the relationship of health anxiety with outcomes including depression, anxiety, annual visits, and physical distress were tested by PROCESS macro (Hayes, 2018) using SPSS (version 19.0). In estimating the moderation effect, health anxiety and patient enablement were mean-centered. Age, gender (male coded as “1,” female as “0”), income (monthly household income less half of the territory-wide median of HKD 10,000 or USD 1,282 coded as “1,” not coded as “0”), education (secondary or more coded as “1” versus less than secondary coded as “0”). To control for other aspects of service experience that may determine overall distress and service utilization, such as the hardware and logistical aspects, overall service satisfaction was also added as a covariate alongside demographic variables. Significant moderation effects were scrutinized by visualizing the effects of health anxiety on the outcomes at +1 *SD*, mean, and −1 *SD* of patient enablement. As the survey was cross-sectional, list-wise deletion was implemented in estimating the moderation models.

## Results

### Participants’ Characteristics

Participants’ characteristics were summarized in Table 1. About three quarters (77.6%) of the participants were female, and the median age was 48.2 (*SD* = 13.3, range = 18–83). Median monthly household income bracket was HKD 13,000–15,999 (USD 1666–2051), which was lower than the territory-wide figure of HKD 20,000 (USD 2564) in 2012. Most participants were married (69.6%); 19.6% had an education level of primary or less, and 80.4% had secondary education or more. In addition, 33.5% of participants were homemakers, 44.9% were working either full-time or part-time, 67.2% of participants had 0–10 annual clinic visits, 28.2% had 11–50 annual clinic visits, and 4.6% had more than 50 annual clinic visits.

**TABLE 1:** Descriptive statistics among key variables.

Variables	Mean	SD	Reliability	*N*	%
Patient enablement (0–12)	5.88	2.83	0.889	609	–
Satisfaction (0–10)	7.80	1.95	–	618	–
Health anxiety (0–7)	3.72	2.06	0.735^a^	631	–
Physical distress (0–10)	2.46	1.92	0.909	628	–
Anxiety (0–21)	5.29	4.97	0.930	628	
0–4 (Minimal)				338	53.8
5–9 (Mild)				174	27.7
10–14 (Moderate)				81	12.9
15–21 (Severe)				35	5.6
Depression (0–27)	5.62	4.89	0.867	632	
0–4 (Minimal)				324	51.3
5–9 (Mild)				188	29.7
10–14 (Moderate)				84	13.3
15–19 (Moderately severe)				22	3.5
20–27 (Severe)				14	2.2

*Parentheses denote possible range of scores of each instrument. ^*a*^Reliability of the Whiteley-7 Index is measured by Kuder-Richardson 20 due to binary response items.*

### Descriptive Statistics and Association Among Variables

Correlational matrix (Table 2) shows that physical distress, depression, and anxiety were strongly correlated (*r*s = 0.55–0.71, *p*s < 0.001), and health anxiety correlated with physical distress, depression, and anxiety (*r*s = 0.47–0.54, *p*s < 0.001). Patient enablement was not correlated with health anxiety but positively with satisfaction, *r* = 0.36, *p* < 0.001, and negatively with depression, *r* = −0.11, *p* = 0.009. Satisfaction was also uncorrelated with health anxiety, but negatively with depression, *r* = −0.09, *p* = 0.032. However, both negative correlations with depression were regarded as weak.

**TABLE 2:** Inter-correlations among key variables.

Variables	A	B	C	D	E
Patient enablement	–				
Satisfaction	0.367**	–			
Health anxiety	0.020	–0.019	–		
Physical distress	–0.053	–0.020	0.474**	–	
Anxiety	–0.069	–0.022	0.539**	0.550**	–
Depression	−0.106**	−0.087*	0.502**	0.618**	0.710**

*p < 0.050; **p < 0.010.

### Associations Between Demographic and Other Variables

Respondents’ well-being measures, including depression, anxiety, health anxiety, patient enablement, physical distress, and annual visits, differed based on gender, age, income, and education. Compared to male respondents, female respondents had higher physical distress, *t*(240) = 4.22, *p* < 0.001, and depression, *t*(625) = 2.51, *p* < 0.013. Age was positively correlated with patient enablement, *r* = 0.12, *p* = 0.004; health anxiety, *r* = 0.17, *p* < 0.001; physical distress, *r* = 0.08, *p* = 0.041; and annual visits, *r* = 0.15, *p* < 0.001, although the magnitudes were considered weak. Moreover, the lower income group reported higher health anxiety, *t*(628) = −4.00, *p* < 0.001; anxiety, *t*(625) = −3.83, *p* < 0.001; physical distress, *t*(625) = −4.76, *p* < 0.001; depression, *t*(629) = −4.58, *p* < 0.001; and annual visits, *t*(211) = −2.62, *p* = 0.009. Last, those with less than a secondary level of education suffered from more depression, *t*(170) = 3.62, *p* < 0.001; anxiety, *t*(176) = 3.51, *p* < 0.001; physical distress, *t*(177) = 4.17, *p* < 0.001; and health anxiety, *t*(623) = 4.68, *p* < 0.001, and were more likely to have more annual visits, *t*(134) = 2.31, *p* = 0.005. Other comparisons were statistically non-significant.

### Moderating Effect of Patient Enablement

**FIGURE 1:**
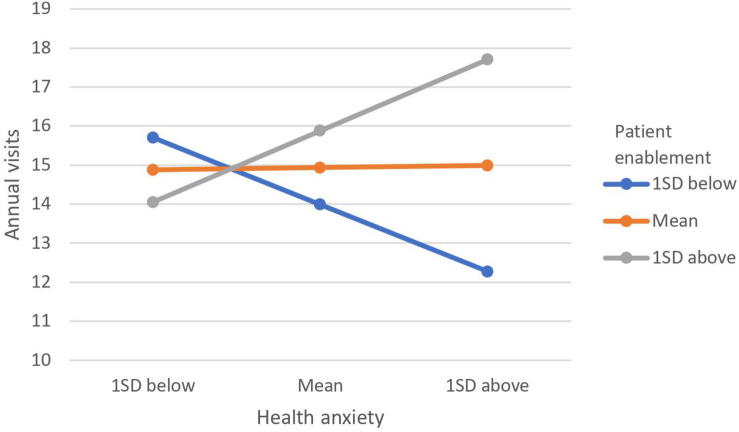
Moderating effect of patient enablement on relationship between health anxiety and annual visits. Moderating effect was illustrated by the levels of annual visits at 1 standard deviation (SD) above mean, at mean and 1 SD below mean for patient enablement and health anxiety.

**FIGURE 2:**
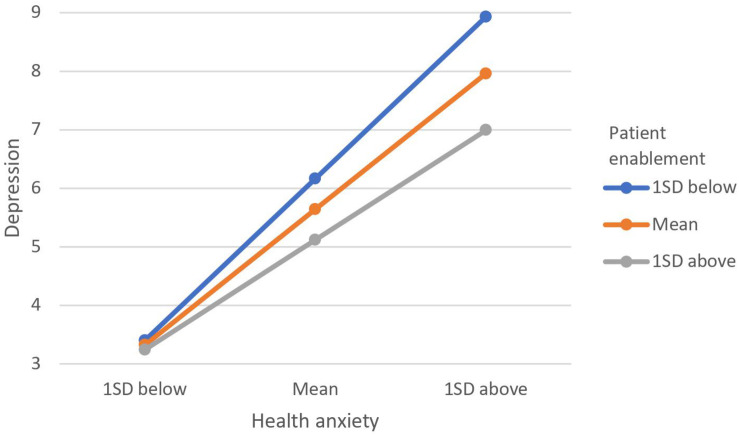
Moderating effect of patient enablement on relationship between health anxiety and depression. Moderating effect was illustrated by the levels of depression at 1 standard deviation (SD) above mean, at mean and 1 SD below mean for patient enablement and health anxiety.

**FIGURE 3:**
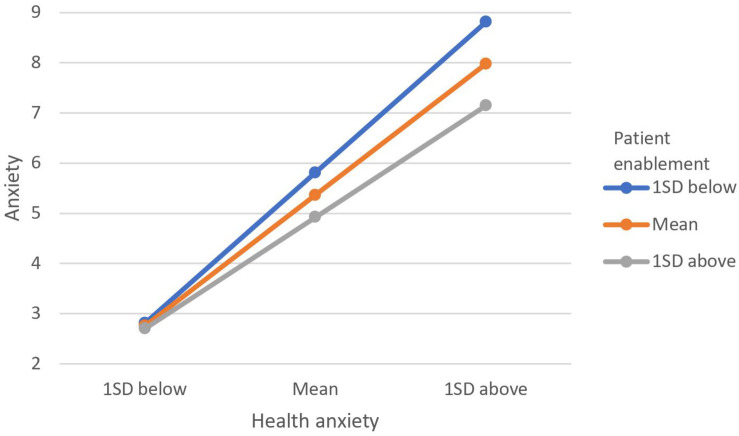
Moderating effect of patient enablement on relationship between health anxiety. Moderating effect was illustrated by the levels of anxiety at 1 standard deviation (SD) above mean, at mean and 1 SD below mean for patient enablement and health anxiety.

Table 3 illustrates the results of the moderation analyses with anxiety, depression, physical distress, and annual visits as the outcome variables. Inspection of the product term (patient enablement × health anxiety) shows that patient enablement was a significant moderator in the relationship between health anxiety with anxiety, depression, and annual visits but not with physical distress. For the relationship between health anxiety and annual visits (Figure 1), individuals with higher patient enablement experienced a positive relationship, and those with lower patient enablement experienced a negative relationship. That is, among people with higher patient enablement, greater health anxiety leads to more visits, and in those with lower patient enablement, higher health anxiety was related to fewer visits. The trends for both depression and anxiety were similar (Figures 2, 3). The positive relationships of health anxiety with depression and anxiety were more salient among individuals with lower patient enablement. In other words, patients with higher patient enablement experienced a weaker relationship between health anxiety and psychological distress (depression and anxiety).

**TABLE 3:** Moderating effect of patient enablement.

	Anxiety (*N* = 584)		Depression (*N* = 585)		Physical distress (*N* = 584)		Annual visits (*N* = 516)	
				
	B	SE	B	SE	B	SE	B	SE
Male^+^	–0.1583	0.4143	–0.7198	0.4093	−0.5366**	0.1625	–1.6758	2.2044
Age	−0.0743**	0.0145	−0.0565**	0.0143	–0.0057	0.0057	0.1500	0.0770
Low income^+^	0.9451*	0.4007	1.0173*	0.3966	0.4220**	0.1569	4.6500*	2.1344
Service satisfaction	0.0947	0.0955	–0.0619	0.0946	0.0118	0.3150	0.8928	0.5083
Secondary education or more	−1.2871**	0.4671	−1.3453**	0.4626	–0.3432	0.1830	–3.0010	2.5261
Health anxiety (H)	1.2777**	0.0861	1.1337**	0.0851	0.3955**	0.0337	0.0269	0.4665
Patient enablement (P)	−0.1576*	0.0665	−0.1862**	0.0659	–0.0432	0.0261	0.3389	0.3600
Interaction (H × P)	−0.0676*	0.0294	−0.0773**	0.0291	–0.0176	0.0115	0.3095*	0.1561
Adjusted *R*^2^	0.330**		0.306**		0.259**		0.055**	

*p < 0.050; **p < 0.010; ^+^Categorical variable. Intercepts were included in the models but not reported here.

## Discussion

The current study examined how patient enablement is correlated with other physical and psychological health indices and how it modulated the impact of health anxiety on psychological distress and treatment-seeking behavior. Replicating earlier findings (e.g., Tolvanen et al., 2017), we found that patient enablement is positively correlated with patient satisfaction. Also, our findings additionally suggest a moderating role of patient enablement, such that highly enabled patients tended to experience a weaker positive relationship between health anxiety and psychological distress (in terms of both anxiety and depression) but a stronger positive association between health anxiety and annual clinic visits. Thus, highly enabled patients, relative to less enabled ones, showed a blunted effect of health anxiety on psychological well-being. At the same time, they also exhibit greater treatment seeking in TCM driven by health anxiety.

Patients tend to use TCM for treating milder symptoms, mollifying the side effects of Western medicine, curing chronic diseases or conditions that are not completely cured by Western medicine (i.e., “clearing the root of the disease”) as well as for health maintenance and tonic care purpose (Lam, 2001; Critchley et al., 2005; Chung et al., 2014). Chung et al. (2009) revealed a group of aging patients without non-communicable diseases who use TCM as an exclusive form of health care. The higher level of health anxiety and poorer psychological well-being documented among the current sample compared to the general population seems to be in line with these previous findings. The use of TCM for health maintenance or clearing the roots of disease could be fueled by the preoccupation with health issues (possibly without a biological impetus) and heightened vigilance toward bodily symptoms. Patients may also somaticize their psychological distress (Cheung et al., 1981; Parker et al., 2001). Symptoms of somatization, such as fatigue, sleep disturbances, and pains could be understood as chronic, mild ailments, which people think would be better managed using TCM rather than Western medicine (Lam, 2001; Critchley et al., 2005; Chung et al., 2014). A study on Chinese Americans in Los Angeles found that somatizers are inclined to perceive their health as poor and utilize both Western and indigenous Chinese medicine (Mak and Zane, 2004). Further studies are warranted to elucidate the association between health anxiety, somatization, and use of TCM or CAM in comparison with mainstream Western medicine.

The current study elucidated the moderating effect of patient enablement. Specifically, among patients who felt more enabled after the consultation, the extent to which health anxiety negatively impacted upon their psychological distress was reduced, whereas, for those who felt less enabled, the burden of health anxiety was not lifted as effectively. That patient enablement was associated with better psychological well-being is in line with our hypothesis. Healthcare professionals have an ethical duty to provide the patient sufficient information about the disease and prognosis, whereas a high-quality consultation should also include suggestions of strategies in coping with illnesses and empowerment of patients’ self-efficacy in self-care and treatment adherence. Information imparted by the clinicians, explicitly or implicitly, may induce a sense of control in patients, enable them to put things in perspective, and diminish the impact of health anxiety on their overall psychological distress. Contrary to our expectation, patient enablement did not moderate the relationship between health anxiety and physical distress. One possibility might be that our sample of primary care patients presented with relatively “pure” cases of health anxiety; that is, their preoccupation with health-related worries may not be based off of the presence of physical symptoms. Although it is more commonly the case that individuals present with concurrent physical health symptoms and health anxiety, health anxiety can exist in isolation of physical symptoms in the general population (Lee et al., 2015).

Our additional analyses found a moderating effect by patient enablement on the relationship between health anxiety and number of annual clinic visits with more enabled patients displaying a stronger link between health anxiety and annual visits. We have also found a positive association between patient enablement and satisfaction with this sample. It is possible that the more enabled the patients, the more they are being reinforced by their high service satisfaction. This may, in turn, cause them to perceive seeking TCM treatment as a legitimate and effective means to deal with their health worries. Again, because we have not found that enablement reduced health anxiety, although it is associated higher satisfaction, highly enabled patients may have learned to take a more active role in seeking medical services in CAM or TCM, in addition to Western medicine, to cope with their perceived health issues.

It is also possible that the moderating effect of patient enablement found in the present study is specific to treatment seeking in TCM as it is stereotypically seen as complementary or alternative to mainstream Western medicine and, therefore, entailing greater voluntary investigation and engagement on the part of the patient in seeking such alternative forms of treatment than simply being a passive recipient of medical care as it is stereotypically the case in Western medicine. Further studies are needed to explicate the relationship between patient enablement and treatment-seeking behavior in the context of both Western medicine as well as TCM and how the patient enablement process should be implemented to facilitate self-management and self-care among patients receiving Western medicine and TCM. It is also of interest to see if different levels of patient enablement are associated with different patterns of treatment choices, especially whether more highly enabled patients would be more inclined to seek treatment with CAM or TCM in addition to mainstream Western medicine, relative to less enabled patients.

### Limitations

Cross-sectional analysis does not establish moderation pathways. Without longitudinal and experimental studies, the direction of moderating effects cannot be confirmed. Inherent in consecutive sampling is self-selecting bias. That we did not record refusal rate and the profile of those who did not participate in the study was also a shortcoming that limits the generalizability of the findings. The survey also only measured respondents’ level of satisfaction toward a single consultation. Further studies are encouraged to replicate the current findings with information from multiple consultations.

### Practical Implications

Cognitive behavioral therapy (CBT) and medication is recommended for severe health anxiety (Barsky and Ahern, 2004; Harding et al., 2008), and other approaches, such as psycho-education (Buwalda and Bouman, 2008) and mindfulness-based intervention (Lovas and Barsky, 2010; Williams et al., 2011), have been tested. A Cochrane review (Thomson and Page, 2007) found that, although CBT is effective in reducing health anxiety and psychological distress, there was less evidence supporting the effectiveness of other approaches. If our current findings are confirmed by further longitudinal and experimental studies, the implication would be that, rather than directly addressing health anxiety with specialized counseling or psychiatric service, healthcare providers may borrow the experiences in CBT programs and focus on helping patients cope with their illness experience and limit its impact on their psychological well-being.

## Conclusion

This study was the first one to investigate whether patient enablement has any effect on health anxiety and its impact on a patient’s psychological well-being. Replicating earlier findings, we found that patient enablement has a significant positive correlation with service satisfaction. We also found that there was a moderating effect of patient enablement in the relationship between health anxiety and psychological distress and treatment seeking in TCM, such that, among highly enabled patients, the relationship between health anxiety and psychological distress is weakened while health anxiety is conducive to treatment-seeking behavior in TCM settings.

## Data Availability Statement

The raw data supporting the conclusions of this article will be made available by the authors, without undue reservation, to any qualified researcher.

## Ethics Statement

The study was approved by the Institutional Review Board of The University of Hong Kong/Hospital Authority Hong Kong West Cluster (HKU/HA HKW IRB). The reference number is UW 13-088. The participants gave written consent to participate in the study.

## Author Contributions

CHC and CLC conceived and conceptualized the study, and obtained the funding for the study. BL and TC performed the data analysis. CHC, BL, TC, HL, GS, and CLC wrote the manuscript. CHC and CLC obtained the funding for the study. All authors have read and approved the manuscript.

## Conflict of Interest

The authors declare that the research was conducted in the absence of any commercial or financial relationships that could be construed as a potential conflict of interest.

## Abbreviations

CAM: Complementary and alternative medicine
TCM: Traditional Chinese medicine.
